# Association of Adult Height with Cardiovascular Mortality: A Systematic Review and Meta-Analysis of Cohort Studies

**DOI:** 10.1155/2022/6959359

**Published:** 2022-10-26

**Authors:** Obaidullah Fahim, Sina Naghshi, Zainab Khademi, Ahmad Esmaillzadeh

**Affiliations:** ^1^Department of Community Nutrition, School of Nutritional Sciences and Dietetics, Tehran University of Medical Sciences, Tehran, Iran; ^2^Department of Nutrition, Faculty of Public Health, Kabul University of Medical Science, Kabul, Afghanistan; ^3^Students' Scientific Research Center, Tehran University of Medical Sciences, Tehran, Iran; ^4^Department of Clinical Nutrition, School of Nutritional Sciences and Dietetics, Tehran University of Medical Sciences, Tehran, Iran; ^5^Department of Public Health, Sirjan School of Medical Sciences, Sirjan, Iran; ^6^Obesity and Eating Habits Research Center, Endocrinology and Metabolism Molecular—Cellular Sciences Institute, Tehran University of Medical Sciences, Tehran, Iran; ^7^Department of Community Nutrition, School of Nutrition and Food Science, Isfahan University of Medical Sciences, Isfahan, Iran

## Abstract

**Background:**

Epidemiological studies on the association between adult height and cardiovascular disease (CVD) mortality have provided conflicting findings. We examined the association between adult height and the risk of CVD mortality.

**Methods:**

We searched PubMed, Scopus, ISI Web of Knowledge, and Google Scholar for relevant studies published up to September 2021. Prospective cohort studies that reported the risk estimates for death from CVD, coronary heart disease (CHD), and stroke were included. The random-effects model was used to calculate summary relative risks (RRs) and 95% confidence intervals (CIs) for the highest vs. lowest categories of adult height.

**Results:**

In total, 20 prospective cohort publications were included in this systematic review and 17 in the meta-analysis. During 5 to 41 years of follow-up, the total number of deaths from CVD was 95,197 (51,608 from CHD and 20,319 from a stroke) among 2,676,070 participants. The summary RR comparing the highest and lowest categories of height was 0.80 (95% CI: 0.74–0.87, *I*^2^ = 59.4%, *n* = 15 studies) for CVD mortality, 0.82 (95% CI: 0.74–0.90, *I*^2^ = 70.6%, *n* = 12) for CHD mortality, 0.73 (95% CI: 0.67–0.80, *I*^2^ = 0%, *n* = 10) for stroke mortality, 0.70 (95% CI: 0.61–0.81, *I*^2^ = 0%, *n* = 4) for hemorrhagic stroke mortality, and 0.88 (95% CI: 0.72–1.08, *I*^2^ = 0%, *n* = 4) for ischemic stroke mortality.

**Conclusion:**

The present comprehensive meta-analysis provides evidence for an inverse association between adult height and the risk of CVD, CHD, and stroke mortality.

## 1. Introduction

Cardiovascular disease (CVD) is now a public health crisis, affecting millions of people in both developed and developing countries. It is one of the most common causes of death [1]. Although obesity is a well-known risk factor for CVD [2], increasing evidence suggests that other body size indicators are involved in CVD development and its mortality. Adult height, which is determined by both genetic and environmental factors, is considered a surrogate marker for in utero and childhood conditions [3]. Although adult height is not a modifiable risk factor, it can provide insights into patterns of early determinants of major diseases of later life. Therefore, studying height would be informative to identify the effects of early diet on CVD risk in later life.

Taller adult height was associated with a reduced risk of all-cause mortality and an increased risk of cancer mortality. However, epidemiological studies on the association between adult height and CVD mortality have provided conflicting findings. Higher height was associated with an increased risk of CVD mortality in a study among females [4], while others reported an inverse association [5–7] or no clear significant association [8, 9]. A recent individual participant meta-analysis showed that adult height was inversely associated with the risk of death from circulatory diseases such as coronary disease, stroke, and heart failure [10]. However, several large relevant studies have been published since the release of that meta-analysis and have reported conflicting results [4, 5, 11–12]. To combine these findings and reach a definite conclusion, we conducted the current comprehensive systematic review and meta-analysis to summarize the available data on the association between adult height and CVD mortality.

## 2. Methods

We followed the Preferred Reporting Items for Systematic Reviews and Meta-Analysis (PRISMA) guidelines for reporting the present meta-analysis.

### 2.1. Search Strategy

We carried out a systematic literature search using online databases, including PubMed, Scopus, ISI Web of Knowledge, and Google Scholar, to identify relevant papers published up to September 2021. No language or time limitations were performed. In the search strategy, a combination of MeSH (medical subject heading terms) and non-MeSH terms was used (Supplementary Table 1). References from all relevant articles were checked to not miss any study.

### 2.2. Eligibility Criteria

Two of the authors (OF and ZK) screened the titles and abstracts of all studies found in the systematic search to identify studies that met our criteria for inclusion in the meta-analysis. Studies were selected if they (1) had a prospective observational design; (2) were conducted on the adult population; and (3) reported relative risks, including risk ratios (RRs), hazard ratios (HRs), and odds ratios (ORs) with 95% confidence intervals (CIs) to determine the association of height with the risk of CVD, coronary heart disease (CHD), and stroke mortality. If several publications were reported from a single cohort, the most comprehensive one was included.

### 2.3. Exclusion Criteria

We excluded letters, comments, reviews, meta-analyses, and ecological studies. We also excluded studies that were conducted on children or adolescents and publications that reported RRs for cardiovascular and respiratory disorder mortality combined.

### 2.4. Data Extraction

After the study selection process, two reviewers (OF and SN) independently extracted data from the original cohort studies. The following items were extracted from each eligible study: the first author's name, publication year, cohort name, country, age range or mean age of study participants, gender, sample size, number of CVD deaths, follow-up duration, height assessment methods, comparison categories, adjusted RR with 95% CI for CVD mortality, and covariates adjusted in the statistical analysis. The greatest degree of adjustment from each study was considered and used for the meta-analysis. Any disagreements between the reviewers were discussed and resolved by the third reviewer (AE).

### 2.5. Quality Assessment of Studies

The Newcastle-Ottawa scale was used to assess the quality of each study. This tool consists of several parts, including the selection of participants (4 scores), comparability (2 scores), and ascertainment of exposure or outcome (3 scores). The total score of NOS ranges between 0 and 9. Studies achieving nine points were considered to provide the highest quality.

### 2.6. Patient and Public Involvement

No patients were involved in the design, conduct, reporting, or dissemination plans of our research.

### 2.7. Statistical Methods

ORs, RRs, and HRs (and 95% CIs) for comparison of the highest and lowest categories of adult height were used to calculate log RR, OR, and HR ± SE. The analyses were performed with the use of a random-effects model to account for between-study heterogeneity. We assessed heterogeneity between studies using Cochran's *χ*^2^ test and quantified the inconsistencies using the *I*^2^ statistic. For the *I*^2^ statistic, we considered the *I*^2^ values of 25%, 50%, and 75% as low, moderate, and high between-study heterogeneity, respectively [13]. We also used the method of Hamling and colleagues [14] to convert risk estimates when the lowest category was not the reference category.

Two studies [15, 16] reported only risk estimates but not corresponding 95% confidence intervals. We calculated 95% confidence intervals by using *P* values and effect estimates [17]. When studies reported risk estimates for CHD and stroke but not for CVD mortality, we pooled risk estimates for CHD and stroke using a fixed-effects model. Then, the pooled risk estimate was included in the meta-analysis of CVD mortality. In case of finding a significant between-study heterogeneity, we performed stratified analyses to explore possible sources of heterogeneity. Between-subgroup heterogeneity was examined through a fixed-effects model. If ≥10 studies were available, we examined the possibility of publication bias by inspecting funnel plots and conducting Egger's and Begg's tests [18]. In case of a significant publication bias, the trim-and-fill method was used to detect the effect of missing studies on the overall effect of meta-analysis. The influence of each individual study on the summary RR was examined by excluding the study in turn. Statistical analyses were conducted using STATA version 14.0. *P* < 0.05 was considered statistically significant for all tests, including Cochran's *Q* test.

## 3. Results

### 3.1. Literature Search

**Figure 1:**
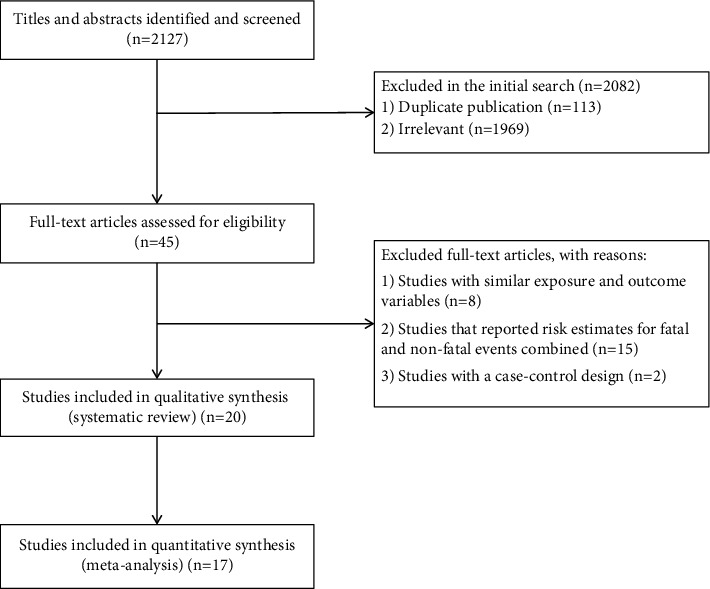
Flow diagram of study selection.

The flow diagram of study selection is shown in Figure 1. Our initial search identified 2127 publications. After excluding duplicates and papers that did not meet the inclusion criteria, 45 remaining papers seemed to be relevant for this meta-analysis. After examination of full texts, an additional 25 papers were excluded: fifteen that reported risk estimates for fatal and nonfatal CVD events combined, eight that were conducted on the same populations with the same outcomes, and two with a case-control design. Finally, 20 publications were included in the systematic review [4–12, 15, 16, 19–27], and 17 were included in the meta-analysis [4–8, 10–12, 15, 20–27].

### 3.2. Characteristics of the Included Studies

Characteristics of the 20 included articles are shown in Supplemental Table 2. All the included articles were published between 1994 and 2021. The number of participants in these studies varied from 1441 to 1,085,949, with an age range between 14 and 99 years. The duration of follow-up time ranged from 5 to 41 years, and the total number of CVD deaths was 95,197 (51,608 from CHD and 20,319 from stroke) among 2,676,070 participants.

Among the 20 included articles, six included only men [5, 7, 8, 20, 21, 24], one included only women [6], and twelve had reported risk estimates for men and women separately [4, 10–12, 15, 16, 19, 22, 23, 25–27]. In total, three papers described studies conducted in the United States [7, 15, 23], sixteen in non-US countries [4–6, 8, 10–12, 16, 19–22, 24–27], and one publication described studies conducted in US and non-US countries [9]. In three publications, height was obtained from a self-reported questionnaire, in sixteen articles, height was measured, and one publication described studies in which both approaches were applied. Most included publications had adjusted for some potential confounders, including body mass index (BMI) (*n* = 16), smoking (*n* = 18), alcohol consumption (*n* = 11), and physical activity (*n* = 8). Based on the NOS score [28], all publications, which had a total score above the median (≥7), were defined as high quality (Supplemental Table 3).

### 3.3. Findings from the Meta-Analysis

#### 3.3.1. Adult Height and Total CVD Mortality

**Figure 2:**
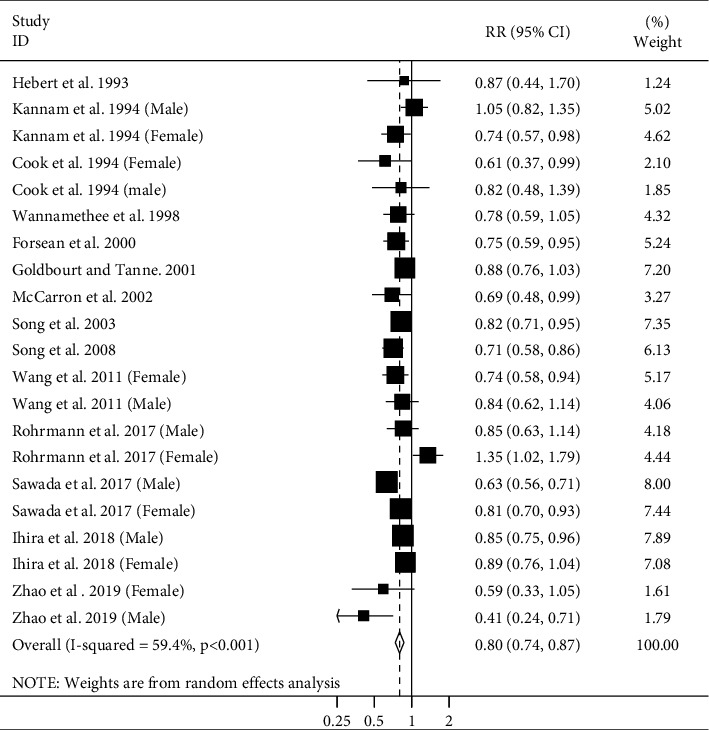
Forest plot for the association between adult height and CVD mortality by considering the highest and lowest height. Horizontal lines represent 95% CIs. Diamonds represent the pooled estimates from the random-effects analysis. RR: relative risk; CI: confidence interval.

Fifteen cohort studies (14 publications) [4–8, 10–12, 15, 20–21, 23, 24, 27] investigated the association between adult height and CVD mortality (stroke and CHD combined). These studies included a total of 1510355 participants with 28955 CVD deaths. Combining RRs from these studies, comparing the highest and lowest categories of adult height, a significant inverse association was found between adult height and risk of CVD mortality (pooled RR: 0.80, 95% CI: 0.74–0.87), with a high heterogeneity among studies (*I*^2^ = 59.4%; *P*_heterogeneity_<0.001) (Figure 2). Publication bias was not evident with Egger's test or Begg's test.

#### 3.3.2. Adult Height and CHD Mortality

**Figure 3:**
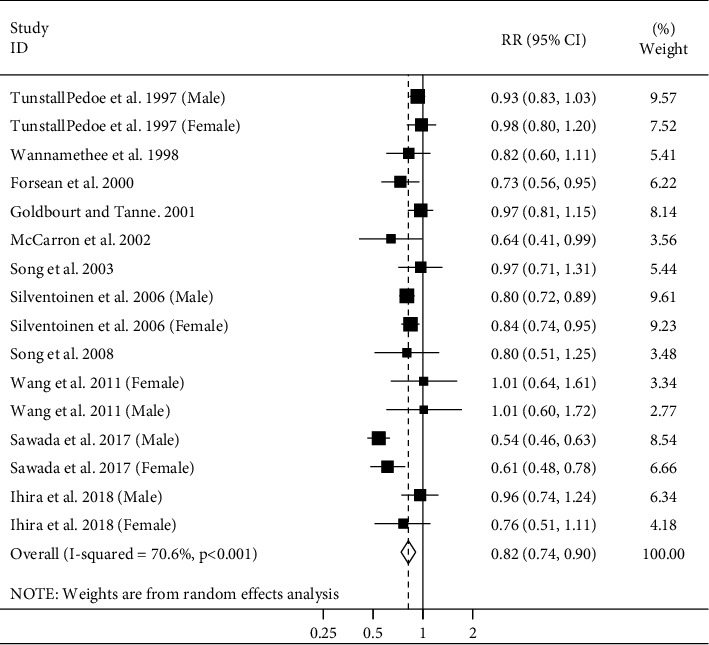
Forest plot for the association between adult height and CHD mortality by considering the highest and lowest height. Horizontal lines represent 95% CIs. Diamonds represent the pooled estimates from the random-effects analysis. RR: relative risk; CI: confidence interval.

In total, 12 studies (11 publications) were included in this meta-analysis [4–6, 8, 12, 20, 21, 24–27]. These studies included a total of 1439521 participants and 16,484 CHD deaths. Considering the highest versus lowest categories of adult height, a significant inverse association was seen between adult height and CHD mortality (pooled RR: 0.82; 95% CI: 0.74–0.90), with a significant between-study heterogeneity (*I*^2^ = 70.6%; *P*_heterogeneity_<0.001) (Figure 3) There was no evidence of small study bias using Egger's test or Begg's test.

#### 3.3.3. Adult Height and Stroke Mortality

**Figure 4:**
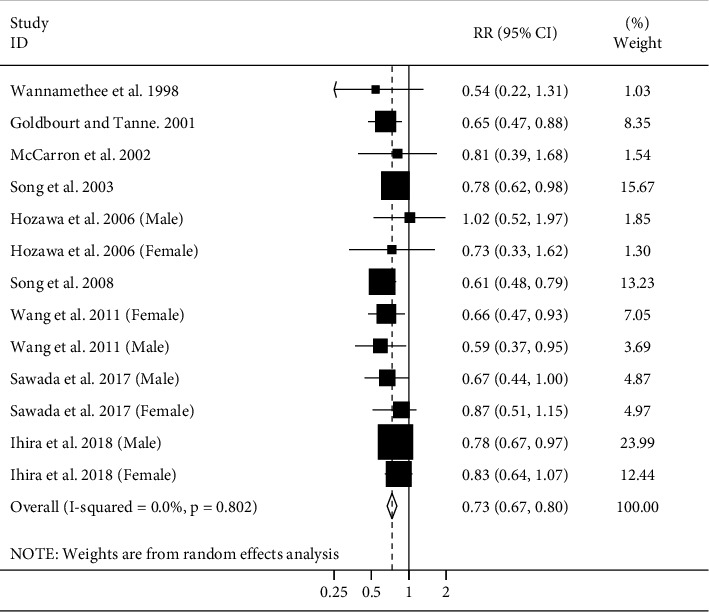
Forest plot for the association between adult height and stroke mortality by considering the highest and lowest height. Horizontal lines represent 95% CIs. Diamonds represent the pooled estimates from the random-effects analysis. RR: relative risk; CI: confidence interval.

Ten cohort studies (nine publications) [4–6, 8, 12, 21, 22, 24, 27] were included in the analysis of adult height and stroke mortality. These studies included a total of 1435375 participants; of them, 7322 died due to stroke. Combining data from these studies indicated a significant inverse association between adult height and risk of stroke mortality (pooled RR: 0.73, 95% CI: 0.67–0.80) (Figure 4). There was no evidence of heterogeneity among studies (*I*^2^ = 0%; *P*_heterogeneity_ = 0.80). We found no substantial publication bias by using Egger's test and Begg's test.

#### 3.3.4. Adult Height and Ischemic Stroke Mortality

**Figure 5:**
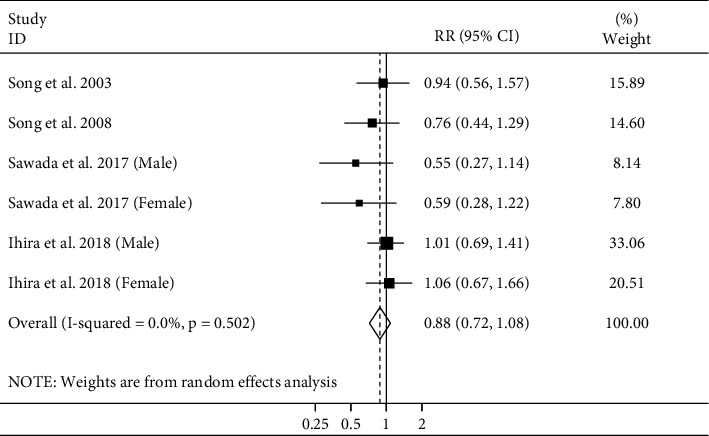
Forest plot for the association between adult height and risk of ischemic stroke mortality by comparing the highest and lowest height. Horizontal lines represent 95% CIs. Diamonds represent the pooled estimates from the random-effects analysis. RR: relative risk; CI: confidence interval.

Four studies [4–6, 12] investigated the association between height and ischemic stroke mortality, with a total of 1248688 participants and 1280 deaths. The summary RR for ischemic stroke mortality, comparing the highest and lowest categories of adult height, was 0.88 (95% CI: 0.72–1.08), indicating no clear significant association between height and ischemic stroke mortality (Figure 5). There was no evidence of heterogeneity between the studies (*I*^2^ = 0.0%; *P*_heterogeneity_ = 0.50). Publication bias tests were not performed (<10 studies).

#### 3.3.5. Adult Height and Hemorrhagic Stroke Mortality

**Figure 6:**
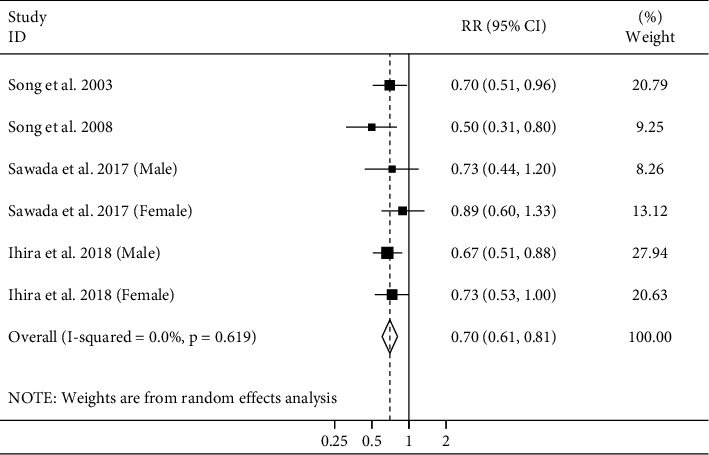
Forest plot for the association between adult height and risk of hemorrhagic stroke mortality by comparing the highest and lowest height. Horizontal lines represent 95% CIs. Diamonds represent the pooled estimates from the random-effects analysis. RR: relative risk; CI: confidence interval.

Four studies [4–6, 12] examined the association between adult height and hemorrhagic stroke mortality. These studies included a total of 1248688 participants and 2646 deaths. Combining data from these studies indicated a significant inverse association between height and hemorrhagic stroke mortality (pooled RR: 0.70, 95% CI: 0.61–0.81) (Figure 6). No heterogeneity of effect estimates on relative risks was observed (*I*^2^ = 0.0%; *P*_heterogeneity_ = 0.61). Publication bias tests were not performed (<10 studies).

### 3.4. Subgroup and Sensitivity Analyses

Supplemental Table 4 shows findings from the different subgroup analyses. In terms of the heterogeneity observed for the association between adult height and CVD mortality, subgroup analyses showed that location of study conduction, exposure assessment method, and adjustment for alcohol consumption might be involved. Additionally, a significant inverse association was observed between adult height and CVD mortality in most subgroup analyses. In terms of CHD mortality, method of exposure assessment and follow-up duration appeared to be the main sources of heterogeneity. Moreover, a significant inverse association was seen in most subgroup analyses. The sensitivity analysis showed that excluding any single study from the analysis did not appreciably alter the pooled effect sizes.

## 4. Discussion

The summary data from cohort studies indicated that adult height was inversely associated with the risk of cardiovascular mortality. Moreover, we found a significant inverse association between adult height and the risk of death from coronary disease and hemorrhagic stroke, while no clear association was seen with ischemic stroke mortality.

CVD is a leading cause of death and a major global health threat. Although height attainment is generally not under individual control, it is a widely available index reflecting the interaction of genetics and various early life conditions. It may indicate underlying mechanistic pathways relevant to the risk of developing CVD. The current study provides up-to-date and reliable evidence on the association between adult height and CVD mortality.

We found that adult height was inversely associated with CVD mortality. Consistent with our findings, a previously published meta-analysis reported that greater height was associated with a lower risk of all cardiovascular deaths [29]. Our results were also in line with the findings from a large-scale individual data pooling project, in which an inverse linear association was seen between height and CVD risk [9]. A potential biological pathway for this association is a positive association between height with insulin sensitivity and insulin-like growth factor 1 (IGF-1), which predicts a lower risk of CVD [30, 31].

The present study has confirmed that taller individuals have a lower risk of death from coronary disease. In a multilocus Mendelian randomization meta-analysis, taller height was associated with a lower risk of CHD, with possible explanations such as better lung function, lower levels of adiposity, lipid profiles, and blood pressure [32]. The findings of the recent individual participant meta-analysis also demonstrated that adult height was inversely associated with the risk of fatal and non-fatal CHD events [9]. A similar finding was also seen in a meta-analysis that examined the associations between BMI and height during childhood and adolescence with the risk of coronary diseases in adulthood. The investigators reported that height in youth was inversely associated with a lower risk of CHD [33]. Moreover, evidence from another meta-analysis showed that adults within the shortest category had a 50% higher risk of CHD morbidity and mortality than tall individuals [30]. Overall, together with all available studies, our findings provide further evidence of an inverse association between height and CHD mortality. This might be explained by narrow coronary arteries and faster heart rate in short individuals, conditions that are involved in the pathogenesis of arterial occlusive events [34].

In this meta-analysis, taller people had a lower risk of death from stroke, especially hemorrhagic stroke mortality. However, we failed to find an inverse association between adult height and ischemic stroke mortality. Unlike our findings, a meta-analysis of 121 prospective cohort studies showed that adult height was inversely associated with fatal and nonfatal ischemic stroke events. However, this study aligns with our findings reported an inverse relationship between height and hemorrhagic stroke events [9]. The exact explanation for conflicting evidence of the link between adult height and the risk of stroke subtypes is unclear. It is thought that shorter adult height is closely associated with poor nutrition and socio-economic conditions during childhood. It has been suggested that hemorrhagic stroke may be more affected by such conditions than ischemic stroke [35, 36]. Furthermore, the lack of a significant association between height and ischemic stroke mortality in our meta-analysis might be related to the relatively small number of included studies.

Multiple lines of evidence support mechanisms through which taller heights may decrease the risk of CVD mortality. Low socio-economic position in childhood might be considered a possible mechanism for an increased risk of CVD in later life [37, 38]. These conditions in childhood may be reflected in the physical feature of short stature and may lead to an increased future risk of mortality from CVD. Moreover, elevated concentrations of IGF-1 are associated with a lower risk of vascular disease [31, 39]. It has been shown that IGF-1 improves vascular endothelial function and myocardial apoptosis [40, 41]. Additionally, shorter adult height has been associated with increased primary systolic pulse augmentation, leading to increased central aortic pressure [42].

### 4.1. Strengths and Limitations

The large number of participants and deaths, prospective nature of included studies, and consideration of subtypes of CVD mortality are the major strengths of this study. Our meta-analysis also has some limitations that should be considered when interpreting the results. As a meta-analysis of cohort studies, confounding by unmeasured risk factors might have influenced our findings. We found significant heterogeneity in some analyses. The number of studies in stratified analyses was limited to fully explore between-study heterogeneity. Some included studies in the current meta-analysis used self-reported heights rather than measured ones, which might have biased our findings. However, most validation studies have found a strong correlation between self-reported and measured height; nevertheless, some over-reporting of height can occur. Although there was no evidence of publication bias in our analysis, it is still possible that some studies with null findings have remained unpublished. The data on covariates used in primary studies were assessed based on single measurement at the study baseline, and their changes throughout the follow-up were not considered in the original analyses.

## 5. Conclusion

In conclusion, our results indicated that greater height was associated with a lower risk of death from CVD, CHD, and stroke. Well-designed, large prospective studies are required to obtain a better indication of the relationship.

## Data Availability

The datasets generated and/or analyzed during the current study are not publicly available but are available from the corresponding author on reasonable request.

## Supplementary Materials

Supplementary Materials Supplemental Table 1: medical subject headings (MeSH) and non-MeSH keywords used to search relevant publications. Supplemental Table 2: characteristics of the included studies on the association between adult height and risk of cardiovascular mortality. Supplemental Table 3: quality assessment of prospective cohort studies included in the current systematic review and meta-analysis on the association between height CVD mortality based on the Newcastle-Ottawa scale. Supplemental Table 4: stratified analysis on the association of adult height with the risk of mortality from CVD, CHD, and stroke.
