# Supplementary material for: Association of Adult Height with Cardiovascular Mortality: A Systematic Review and Meta-Analysis of Cohort Studies
Source: Int J Clin Pract. 2022 Oct 26;2022:6959359. doi: 10.1155/2022/6959359 (PMC9629919; doi:10.1155/2022/6959359)
Supplement: Supplementary Materials — Supplemental Table 1: medical subject headings (MeSH) and non-MeSH keywords used to search relevant publications. Supplemental Table 2: characteristics of the included studies on the association between adult height and risk of cardiovascular mortality. Supplemental Table 3: quality assessment of prospective cohort studies included in the current systematic review and meta-analysis on the association between height CVD mortality based on the Newcastle-Ottawa scale. Supplemental Table 4: stratified analysis on the association of adult height with the risk of mortality from CVD, CHD, and stroke. [file 6959359.f1.docx]

**Online Supporting Material**

**Supplemental Table 1**: Medical subject headings (MeSH) and non-MeSH keywords used to search relevant publications

| **Concept 1** | "height"[Title/abstract] OR "body Height"[Title/abstract] OR "stature"[Title/abstract] OR "body Height"[Mesh] OR "body height"[Title/abstract] |
| --- | --- |
| **Concept 2** | "mortality"[Mesh] OR "death"[Mesh] OR "survival"[Mesh] OR "mortality"[Subheading] OR "fatal Outcome"[Mesh] OR "mortality"[Title/abstract] OR "fatal"[Title/abstract] OR "survive"[Title/abstract] OR "death"[Title/abstract] OR "survival"[Title/abstract] OR "cardiovascular diseases"[Title/abstract] OR "coronary disease"[Title/abstract] OR "myocardial Ischemia"[Title/abstract] OR "coronary artery disease"[Title/abstract] OR "myocardial infarction"[Title/abstract] OR stroke[Title/abstract] OR "cardiovascular diseases"[Mesh] OR "coronary disease"[Mesh] OR "myocardial Ischemia"[Mesh] OR "coronary artery disease"[Mesh] OR "myocardial infarction"[Mesh] OR "stroke"[Mesh] |

The combination of keywords as mentioned above was used to search online databases.

("concept 1" AND "concept 2")

**Online Supporting Material**

**Supplemental Table 2**: Characteristics of included studies on the association between adult height and risk of cardiovascular mortality

| Author | Country | Age* | Sample size٭ | Follow up (years) | Deaths | Exposure assessment | Median/cutoff point | Mortality outcome | RR (95%CI) | Adjustment |
| --- | --- | --- | --- | --- | --- | --- | --- | --- | --- | --- |
| Wang et al (2011) | China | 40-74 | F: 74869 | 12 | 1018 | Measured | ≥162 vs. <153 | CVD | 0.74 (0.58-0.94) | Birth calendar year, age, education, income, occupation, BMI, WHR, cigarette smoking, alcohol consumption, total physical activity (MET hours/week), daily intake of energy, red meat, fruit and vegetables and menopause status |
|  |  |  |  |  | 568 |  |  | Stroke | 0.66 (0.47-0.93) |  |
|  |  |  |  |  | 222 |  |  | IHD | 1.01 (0.64-1.61) |  |
|  |  |  | M: 61333 | 6 | 602 |  | ≥175 vs. <165 | CVD | 0.84 (0.62-1.14) | Birth calendar year, age, education, income, occupation, BMI, WHR, cigarette smoking, alcohol consumption, total physical activity (MET hours/week), daily intake of energy, red meat, fruit and vegetables |
|  |  |  |  |  | 295 |  |  | Stroke | 0.59 (0.37-0.95) |  |
|  |  |  |  |  | 142 |  |  | IHD | 1.01 (0.60-1.72) |  |
| Ihira et al (2018) | Japan | 40-69 | M:  50755 | 19/1 | 1525 | Self-reported | ≥168 vs. <160 | Heart disease | 0.86 (0.73-1.01) | Public health center, birth year, body mass index, smoking status, alcohol consumption, history of hypertension, history of diabetes and leisure-time sports or physical exercise. |
|  |  |  |  |  | 579 |  |  | MI | 0.96 (0.74-1.24) |  |
|  |  |  |  |  | 1133 |  |  | Cerebrovascular disease | 0.83 (0.69-0.99) |  |
|  |  |  |  |  | 512 |  |  | Hemorrhagic stroke | 0.67 (0.51-0.88) |  |
|  |  |  |  |  | 290 |  |  | Ischemic stroke | 1.01 (0.69-1.47) |  |
|  |  |  | F:  57039 | 20/2 | 920 |  | ≥156 vs. <149 | Heart disease | 0.96 (0.74-1.16) | Public health center, birth year, body mass index, smoking status, alcohol consumption, history of hypertension, history of diabetes (yes or no) and leisure-time sports or physical exercise, menopausal status, and age at menarche. |
|  |  |  |  |  | 291 |  |  | MI | 0.76 (0.51-1.11) |  |
|  |  |  |  |  | 750 |  |  | Cerebrovascular disease | 0.84 (0.66-1.05) |  |
|  |  |  |  |  | 410 |  |  | Hemorrhagic stroke | 0.73 (0.53-1.00) |  |
|  |  |  |  |  | 185 |  |  | Ischemic stroke | 1.06 (0.67-1.66) |  |
| Song et al (2003) | South Korea | 40-64 | M: 386627 | 6 | 1263 | Measured | ≥175 vs. ≤162 | Stroke | 0.78 (0.62-0.98) | Age, diastolic blood pressure, glucose, cholesterol, body mass index, alcohol drinking habits, smoking habits, and exercise, monthly salary, area of residency, occupation. |
|  |  |  |  |  | 636 |  |  | Hemorrhagic stroke | 0.70 (0.51-0.96) |  |
|  |  |  |  |  | 262 |  |  | Ischemic stroke | 0.94 (0.56-1.57) |  |
|  |  |  |  |  | 649 |  |  | CHD | 0.97 (0.71-1.31) |  |
| Forsean et al (2000) | Finland | 40-59 | M: 1441 | 35 | 638 | Measured | <167 vs. >172 | CVD | 1.33 (1.05-1.69) | weight, age, cholesterol, occupation, area, smoking and systolic blood pressure. |
|  |  |  |  |  | 507 |  | <167 vs. >172 | CHD | 1.36 (1.05-1.77) |  |
| Goldbourt and Tanne (2001) | Israel | ≥40 | M: 10059 | 23 | 1092 | Measured | ≤162 vs. ≥172 | CHD | 1.03 (0.87-1.23) | Age |
|  |  |  |  |  | 363 |  |  | Stroke | 1.54 (1.13-2.10) |  |
| Hozawa et al (2006) | Japan | 30-92 | M: 3969 | 19 | 158 | Measured | ≥166 vs. ≤157 | Stroke | 1.02 (0.52-1.97) | Age, weight, systolic BP, diabetes, TC, antihypertensive drug, smoking, drinking |
|  |  |  | F: 4955 | 19 | 132 |  | ≥154 vs. ≤146 | Stroke | 0.73 (0.33-1.62) |  |
| Kannam et al (1994) | U.S.A. | 28-62 | M: 2019 | 35/6 | 574 | Measured | ≤65.75 vs. ≥69.75 | CVD | 0.95 (0.74-1.22) | Age, hypertension, cigarette smoking, serum cholesterol, diabetes, relative weight, and alcohol intake |
|  |  |  | F: 2585 | 35/6 | 473 |  | ≤61 vs. ≥64.5 | CVD | 1.34 (1.02-1.75) |  |
| Rohrmann et al (2017) | Switzerland | 14-99 | M: 16831 | 18/6 | 1375 | Measured | Tallest vs. average | CVD | 0.93 (0.66-1.33) | year of birth, year of survey, BMI; alcohol, smoking, education, marital status, professional status, nationality and language. |
|  |  |  | F: 18654 | 19/3 | 1301 |  | Tallest vs. average | CVD | 1.33 (0.96-1.85) |  |
| Sawada et al (2017) | European | 20-70 | M: 144,962 | 12/5 | 4399 | Measured | <168 vs.≧181 | Circulatory disease | 0.63 (0.56-0.71) | Education level, smoking status, physical activity, alcohol consumption, weight (quintiles), intake of energy. |
|  |  |  |  |  | 2444 |  |  | IHD | 0.54 (0.46-0.63) |  |
|  |  |  |  |  | 1276 |  |  | MI | 0.54 (0.43-0.67) |  |
|  |  |  |  |  | 243 |  |  | Hemorrhagic stroke | 0.73 (0.44-1.20) |  |
|  |  |  |  |  | 109 |  |  | Ischemic stroke | 0.55 (0.27-1.14) |  |
|  |  |  | F: 264,786 |  | 2947 |  | <156 vs. ≧168 | Circulatory disease | 0.81 (0.70-0.93) | Education level, smoking status, physical activity, alcohol consumption, weight (quintiles), intake of energy, menopausal status, and menopausal hormone use. |
|  |  |  |  |  | 1038 |  |  | IHD | 0.61 (0.48-0.78) |  |
|  |  |  |  |  | 563 |  |  | MI | 0.67 (0.49-0.92) |  |
|  |  |  |  |  | 343 |  |  | Hemorrhagic stroke | 0.89 (0.60-1.33) |  |
|  |  |  |  |  | 111 |  |  | Ischemic stroke | 0.59 (0.28-1.22) |  |
| Silventoinen et al (2006) | Denmark, Finland, Sweden | 18-95 | M/F: 74704 | 25-38 | 5943 | Self-reported | Q1 vs. Q4 | CHD | 1.25 (1.12-1.39) | Age, study center, and interaction between age and study center. |
|  |  |  |  |  |  |  | Q1 vs. Q4 | CHD | 1.19 (1.05-1.35) |  |
| Tunstall­Pedoe et al (1997) | Scotland | 40­59 | M: 5754 | 7/6 | 159 | Measured | Q5 vs. Q1 | CHD | 0.93 (0.83-1.03) | Age |
|  |  |  | F: 5875 |  | 47 |  | Q5 vs. Q1 | CHD | 0.98 (0.80-1.20) |  |
| Wannamethee et al (1998) | Britain | 40­59 | M: 7735 | 16/8 | 63 | Measured | <167.7 Vs. >178.9 | Stroke | 0.54 (0.22-1.31) | Age, social class, smoking, preexisting coronary heart disease, stroke, diabetes mellitus, physical activity, alcohol intake, body mass index, cholesterol, antihypertensive treatment, and systolic blood pressure. |
|  |  |  |  |  | 465 |  |  | CHD | 0.82 (0.60-1.11) |  |
| Zhao et al (2019) | China | ≥18 | F: 10448 | 6/01 | 227 | Measured | Q5 vs. Q1 | CVD | 0.59 (0.33-1.05) | Age, income, education level, smoking, drinking, and physical activity at baseline, body mass index, waist circumference, systolic blood pressure, fasting glucose, triglycerides, total cholesterol, and high-density lipoprotein cholesterol at baseline. |
|  |  |  | M: 6815 |  | 257 |  | Q5 vs. Q1 | CVD | 0.41 (0.24-0.71) |  |
| McCarron et al (2002) | Scotland | 16.1-30.0 | M: 8361 | 41/3 | 339 | Measured | >181 vs. <169 | CVD | 0.69 (0.48-0.99) | Systolic blood pressure, body mass index, smoking, father’s social class, and year of birth quintile. |
|  |  |  |  |  | 242 |  |  | CHD | 0.64 (0.41-0.99) |  |
|  |  |  |  |  | 67 |  |  | Stroke | 0.81 (0.39-1.68) |  |
| Smith et al (1999) | Scotland | 45–64 | M: 7052 | 20 | 1281 | Measured | Per 10 cm | CHD | 1.12 (1.03-1.22) | social class, deprivation category, smoking, diastolic blood pressure, cholesterol, body mass index, and FEV score. |
|  |  |  |  |  | 302 |  |  | Stroke | 1.30 (1.10-1.55) |  |
|  |  |  | F: 8354 |  | 771 |  | Per 10 cm | CHD | 1.22 (1.08-1.37) | Social class, deprivation category, smoking, diastolic blood pressure, cholesterol, body mass index, and FEV score. |
|  |  |  |  |  | 386 |  |  | Stroke | 1.20 (1.02-1.43) |  |
| Hebert et al (1993) | USA | 40-84 | M: 22071 | 5 | 164 | Self-reported | ≥73 vs. ≤67 | CVD | 0.87 (0.44-1.70) | Age, aspirin assignment, 1-carotene assignment, body mass index, smoking, history of hypertension, diagnosis of diabetes, history of elevated cholesterol, history of angina pectoris, parental history of Ml prior to age 60, alcohol use, and exercise frequency at least weekly. |
| Jousilahti et al. (2000) | Finland | 25-64 | M: 15163 | 16 | 1601 | Measured | Per 5 Cm | CVD | 0.94 (0.90-0.98) | Age- and birth-cohort, Smoking, Cholesterol, Systolic  blood pressure, Diabetes, Body mass Index, family history of CHD, family history of stroke, Socio-economic status |
|  |  |  |  |  | 1188 |  |  | CHD | 0.96 (0.92-1.00) |  |
|  |  |  |  |  | 234 |  |  | Stroke | 0.84 (0.77-0.92) |  |
|  |  |  | F:16016 |  | 750 |  |  | CVD | 0.94 (0.89-0.99) |  |
|  |  |  |  |  | 428 |  |  | CHD | 0.93 (0.85-1.02) |  |
|  |  |  |  |  | 196 |  |  | Stroke | 0.90 (0.79-1.03) |  |
| Cook et al. 1994 | USA | >65 | M: 1115 | 7 | 378 | Self-reported | ≤63 vs. ≥70 | CVD | 1.22 (0.72-2.07) | Age, body mass index, and smoking |
|  |  |  | F: 1840 |  |  |  | ≤59 vs. ≥65 |  | 1.65 (1.01-2.69) |  |
| Song et al. (2008) | South Korea | 40-64 | F: 344519 | 9.86 | 2402 | Measured | ≥161 vs. <149 | CVD | 0.71 (0.58-0.86) | Age, systolic blood pressure, serum total cholesterol level, fasting blood glucose level, body mass index, cigarette smoking, alcohol consumption, regular exercise, quantile of monthly salary, occupation and area of residence |
|  |  |  |  |  | 408 |  |  | CHD | 0.80 (0.51-1.25) |  |
|  |  |  |  |  | 1521 |  |  | Stroke | 0.61 (0.48-0.79) |  |
|  |  |  |  |  | 502 |  |  | Hemorrhagic stroke | 0.50 (0.31-0.80) |  |
|  |  |  |  |  | 323 |  |  | Ischemic stroke | 0.76 (0.44-1.29) |  |
| The Emerging Risk Factors Collaboration (2012) | Different countries | 55 | M/F: 1085949 | 13.7 |  | Measured/ Self-reported | Per SD | CVD | 0.94 (0.93-0.96) | Age, sex, year of birth and smoking status |
|  |  |  |  |  |  |  |  | CHD | 0.93 (0.91-0.95) |  |
|  |  |  |  |  |  |  |  | Stroke | 0.92 (0.89-0.95) |  |

Abbreviation: RR: Relative Risk - CI: confidence interval- M: male- F: female- BMI: body mass index- US: United States- CVD: cardiovascular diseases- CHD: coronary heart diseases- IHD: Ischemic heart disease- MI: Myocardial Infarction.

*Presented as mean or rang

**Online Supporting Material**

**Supplemental Table 3**: Quality assessment of prospective cohort studies included in the current systematic review and meta-analysis on the association between height CVD mortality based on the Newcastle-Ottawa scale

| Author | Representativeness of the exposed cohort | Selection of the non-exposed cohort | Ascertainment of exposure | Outcome of interest was not present at the start of the study | BMI adjustment | Controls for any additional factor | Assessment of outcome | Follow-up long enough | Adequacy of follow-up of cohorts | Total |
| --- | --- | --- | --- | --- | --- | --- | --- | --- | --- | --- |
| Wang et al (2011) | * | * | * | * | * | * | * |  | * | 8 |
| Ihira et al (2018) | * | * |  | * | * | * | * | * | * | 8 |
| Song et al (2003) | * | * | * | * | * | * | * |  | * | 8 |
| Forsean et al (2000) |  | * | * | * | * | * | * | * | * | 8 |
| Goldbourt and Tanne (2001) | * | * | * | * |  | * | * | * | * | 8 |
| Hozawa et al (2006) | * | * | * | * | * | * | * | * | * | 9 |
| Kannam et al (1994) | * | * | * | * | * | * | * | * | * | 9 |
| Rohrmann et al (2017) | * | * | * | * | * | * | * | * | * | 9 |
| Sawada et al (2017) | * | * | * | * | * | * | * |  | * | 8 |
| Silventoinen et al (2006) | * | * |  | * |  | * | * | * | * | 7 |
| Tunstall­Pedoe et al (1997) | * | * | * | * |  | * | * |  | * | 7 |
| Wannamethee et al (1998) | * | * | * | * | * | * | * | * | * | 9 |
| Zhao et al (2019) | * | * | * | * | * | * | * |  | * | 8 |
| McCarron et al (2002) | * | * | * | * | * | * | * | * | * | 9 |
| Smith et al (1999) | * | * | * | * | * | * | * | * | * | 9 |
| Hebert et al (1993) | * | * |  | * | * | * | * |  | * | 7 |
| Jousilahti et al (2000) | * | * | * | * | * | * | * | * | * | 9 |
| Cook et al (1994) | * | * |  | * | * | * | * |  | * | 7 |
| Song et al (2008) | * | * | * | * | * | * | * |  | * | 8 |
| The Emerging Risk Factors Collaboration (2012) | * | * | * | * |  | * | * | * | * | 8 |

**Online Supporting Material**

**Supplemental Table 4**: Stratified analysis on association of adult height with risk of mortality from CVD, CHD, and stroke

|  | | | **CVD mortality** | | | | |  | | **CHD mortality** | | | | |  | | **Stroke mortality** | | | | |
| --- | --- | --- | --- | --- | --- | --- | --- | --- | --- | --- | --- | --- | --- | --- | --- | --- | --- | --- | --- | --- | --- |
|  | | | **n^1^** | **Pooled RR (95% CI)^2^** | **I^2^ (%)^3^** | **P-heterogeneity^4^** |  | | **n^1^** | | **Pooled RR (95% CI)^2^** | **I^2^ (%)^3^** | **P-heterogeneity^4^** |  | | **n^1^** | | **Pooled RR (95% CI)^2^** | **I^2^ (%)^3^** | **P-heterogeneity^4^** |  |
|  | Location | |  |  |  |  |  | |  | |  |  |  |  | |  | |  |  |  |  |
|  |  | US | 5 | 0.83 (0.68-1.02) | 27.7 | 0.23 |  | | - | | - | - | - |  | | - | | - | - | - |  |
|  |  | Non-US | 16 | 0.79 (0.73-0.87) | 65.1 | <0.001 |  | | 16 | | 0.82 (0.74-0.90) | 70.6 | <0.001 |  | | 13 | | 0.73 (0.67-0.80) | 0 | 0.80 |  |
|  | Sex | |  |  |  |  |  | |  | |  |  |  |  | |  | |  |  |  |  |
|  |  | Male and female | - | - | - | - |  | | - | | - | - | - |  | | - | | - | - | - |  |
|  |  | Male | 13 | 0.79 (0.71-0.88) | 59 | 0.004 |  | | 10 | | 0.82 (0.71-0.94) | 78.2 | <0.001 |  | | 8 | | 0.74 (0.66-0.83) | 0 | 0.81 |  |
|  |  | Female | 8 | 0.81 (0.70-0.93) | 63.3 | 0.008 |  | | 6 | | 0.82 (0.70-0.95) | 48.5 | 0.08 |  | | 5 | | 0.72 (0.62-0.83) | 0 | 0.41 |  |
|  | Follow-up duration | |  |  |  |  |  | |  | |  |  |  |  | |  | |  |  |  |  |
|  |  | >15 years | 10 | 0.87 (0.79-0.96) | 46.5 | 0.05 |  | | 8 | | 0.83 (0.78-0.89) | 2.7 | 0.40 |  | | 7 | | 0.77 (0.68-0.87) | 0 | 0.83 |  |
|  |  | <15 years | 11 | 0.73 (0.66-0.80) | 41.6 | 0.07 |  | | 8 | | 0.82 (0.66-1.01) | 83.8 | <0.001 |  | | 6 | | 0.70 (0.61-0.79) | 0 | 0.58 |  |
|  | Exposure assessment | |  |  |  |  |  | |  | |  |  |  |  | |  | |  |  |  |  |
|  |  | Measured | 16 | 0.79 (0.72-0.87) | 66.1 | <0.001 |  | | 12 | | 0.81 (0.70-0.94) | 77.6 | <0.001 |  | | 11 | | 0.70 (0.62-0.78) | 0 | 0.83 |  |
|  |  | Self-reported | 5 | 0.85 (0.78-0.94) | 0 | 0.71 |  | | 4 | | 0.83 (0.77-0.89) | 0 | 0.59 |  | | 2 | | 0.80 (0.69-0.93) | 0 | 0.70 |  |
|  | Adjustment for energy | |  |  |  |  |  | |  | |  |  |  |  | |  | |  |  |  |  |
|  |  | Yes | 4 | 0.74 (0.63-0.86) | 64.6 | 0.03 |  | | 4 | | 0.70 (0.53-0.93) | 71.1 | 0.01 |  | | 4 | | 0.69 (0.57-0.85) | 0 | 0.62 |  |
|  |  | No | 17 | 0.82 (0.76-0.90) | 47.9 | 0.01 |  | | 12 | | 0.87 (0.82-0.92) | 10.7 | 0.34 |  | | 9 | | 0.74 (0.67-0.82) | 0 | 0.68 |  |
|  | Adjustment for BMI | |  |  |  |  |  | |  | |  |  |  |  | |  | |  |  |  |  |
|  |  | Yes | 20 | 0.79 (0.73-0.86) | 59.9 | <0.001 |  | | 11 | | 0.77 (0.65-0.90) | 64.1 | 0.002 |  | | 12 | | 0.74 (0.67-0.81) | 0 | 0.78 |  |
|  |  | No | 1 | 0.88 (0.76-1.03) |  |  |  | | 5 | | 0.88 (0.82-0.96) | 42.1 | 0.14 |  | | 1 | | 0.65 (0.47-0.88) | - | - |  |
|  | Adjustment for alcohol use | |  |  |  |  |  | |  | |  |  |  |  | |  | |  |  |  |  |
|  |  | Yes | 16 | 0.81 (0.73-0.89) | 67.1 | <0.001 |  | | 9 | | 0.79 (0.65-0.95) | 71 | 0.001 |  | | 11 | | 0.74 (0.67-0.81) | 0 | 0.71 |  |
|  |  | No | 5 | 0.81 (0.72-0.91) | 0 | 0.46 |  | | 7 | | 0.87 (0.80-0.94) | 42.9 | 0.10 |  | | 2 | | 0.67 (0.50-0.90) | 0 | 0.58 |  |
|  | Adjustment for smoking | |  |  |  |  |  | |  | |  |  |  |  | |  | |  |  |  |  |
|  |  | Yes | 21 | 0.80 (0.74-0.87) | 59.4 | <0.001 |  | | 12 | | 0.79 (0.67-0.92) | 70.9 | <0.001 |  | | 13 | | 0.73 (0.67-0.80) | 0 | 0.80 |  |
|  |  | No | - | - | - | - |  | | 4 | | 0.87 (0.80-0.95) | 45.3 | 0.14 |  | | - | | - | - | - |  |
|  | Adjustment for physical activity | |  |  |  |  |  | |  | |  |  |  |  | |  | |  |  |  |  |
|  |  | Yes | 13 | 0.76 (0.70-0.83) | 52.9 | 0.01 |  | | 10 | | 0.77 (0.65-0.92) | 67.6 | 0.001 |  | | 10 | | 0.74 (0.67-0.81) | 0 | 0.71 |  |
|  |  | No | 8 | 0.88 (0.76-1.02) | 57.2 | 0.02 |  | | 6 | | 0.87 (0.81-0.95) | 42.4 | 0.12 |  | | 3 | | 0.71 (0.54-0.93) | 0 | 0.48 |  |

Abbreviation: RR: relative risk- CI: confidence interval- BMI: body mass index- US: united states- CVD: cardiovascular diseases- CHD: coronary heart diseases

^1^Number of effect sizes

^2^Obtained from the random-effects model

^3^Inconsistency- percentage of variation across studies due to heterogeneity

^4^Obtained from the Q-test
